# P53, Somatostatin receptor 2a and Chromogranin A immunostaining as prognostic markers in high grade gastroenteropancreatic neuroendocrine neoplasms

**DOI:** 10.1186/s12885-019-6498-z

**Published:** 2020-01-10

**Authors:** Kirstine Nielsen, Tina Binderup, Seppo W. Langer, Andreas Kjaer, Pauline Knigge, Veronica Grøndahl, Linea Melchior, Birgitte Federspiel, Ulrich Knigge

**Affiliations:** 1 Department of Surgical Gastroenterology C, Copenhagen University Hospital, Rigshospitalet, Copenhagen, Denmark; 2 Department of Clinical Endocrinology, Copenhagen University Hospital, Rigshospitalet, Copenhagen, Denmark; 3 ENETS Neuroendocrine Tumor Centre of Excellence, Copenhagen University Hospital, Rigshospitalet, Copenhagen, Denmark; 4 Department of Clinical Physiology, Nuclear Medicine & PET and Cluster for Molecular Imaging, Department of Biomedical Sciences, Rigshospitalet and University of Copenhagen, Copenhagen, Denmark; 5 Department of Oncology,, Copenhagen University Hospital, Rigshospitalet, Copenhagen, Denmark; 6 Department of Pathology, Copenhagen University Hospital, Rigshospitalet, Copenhagen, Denmark

**Keywords:** Gastroenteropancreatic neuroendocrine neoplasms, p53, Somatostatin receptor 2a, Chromogranin A, Neuroendocrine carcinomas, NEC, NET G3, Survival, Prognostication

## Abstract

**Background:**

High grade gastroenteropancreatic (GEP) neuroendocrine neoplasms (NEN) with a Ki67 proliferation index > 20%, include well-differentiated tumours grade 3 (NET G3) and poorly differentiated (PD) neuroendocrine carcinomas (NEC). Abnormal p53-expression is a feature of PD tumours, while expression of chromogranin A (CgA) and somatostatin-receptor 2a (SSTR-2a) may be a feature of well-differentiated tumours. The aim of this study was to elucidate the expression and prognostic value of these three markers in 163 GEP-NEN patients with a Ki67-index > 20%.

**Method:**

Clinical data, histopathology and overall survival were analysed according to Kaplan-Meier’s method and Cox regression. The expression of SSTR-2a, CgA and synaptophysin was analysed in tumour specimens by immunohistochemistry, and semi-quantitatively scored as negative (< 5%), heterogeneously positive (5–30%) or strongly positive (> 30%). P53 was defined as normal when scored as heterogeneously positive (1–30%), and abnormal when negative (0%) or strongly positive (> 30%).

**Results:**

In multivariate analysis, better survival was observed among patients with heterogeneously positive p53 compared to strongly positive (*p* < 0.001). When dichotomised, tumours with a heterogeneously positive p53 vs. negative and strongly positive p53 also showed a significantly better survival (*p* = 0.002).

Survival was significantly worse for negative CgA compared to heterogeneously positive CgA (*p* = 0.02). Strongly positive SSTR-2a expression was found in 26% of the 163 included patients. Well-differentiated morphology correlated with strong expression of SSTR-2a and CgA, and heterogeneously positive p53-staining, and was more frequent in pancreatic primaries. In pancreatic primaries, strongly positive SSTR-2a was associated with longer survival (univariate analysis, *p* = 0.02). A significantly lower Ki67 proliferation index was found in patients with a heterogeneously positive p53, a positive SSTR-2a and CgA expression.

**Conclusion:**

Our results suggest that abnormal p53-expression is an independent negative prognostic marker in GEP-NEN with a Ki67-index > 20%. Patients with heterogeneously positive p53 had the best prognosis. SSTR-2a was a positive prognostic marker in pancreatic NEN. Negative CgA was associated with a significantly worse OS compared to heterogeneously positive CgA-expression in a multivariate sub-analysis. Lower Ki67 index correlated significantly with heterogeneously positive p53, positive SSTR-2a and CgA expression.

## Background

Gastroenteropancreatic (GEP) neuroendocrine neoplasms (NEN) with a Ki67 proliferation index above 20% are a heterogeneous group of rare, highly malignant neoplasms associated with a poor prognosis [1] Immunohistochemically assessed tumour expression of synaptophysin and/or CgA is mandatory for the NEN diagnosis. CgA is a part of the secretory granules of neuroendocrine cells. Somatostatin receptor (SSTR) expression is a hallmark of NEN biology. Impaired or abnormal tumour suppressor protein p53 expression has been found to be an important marker of aggressive disease in several cancer forms, including GEP-NEC, where abnormal immunohistochemistry (IHC) expression of the p53 protein indicating mutations, or inactivating mutations in the *TP53* gene are the most common molecular alterations observed, regardless of primary site [2].

The WHO 2010 classification divided GEP-NEN according to the Ki67 index (and mitotic count) into neuroendocrine tumours (NET) grade 1 (Ki67 < 2%), NET grade 2 (Ki67 3–20%) and neuroendocrine carcinomas (NEC) grade 3 (Ki67 > 20%). NEC was further subdivided into small and large cell morphology [3].

It has been discussed whether to include tumour differentiation in the classification of NEN G3 [1, 4–7]. The recent WHO 2019 classification of all GEP-NEN extend that tumours with Ki67 > 20% are divided into well differentiated NET G3 and poorly differentiated NEC. Still, all NEC should be divided into small cell (SCNEC) and large cell (LCNEC) [8].

The sub classification into NET G3 and NEC has recently been validated for its prognostic relevance, and survival of patients with NET G3 is significantly better compared to patients with NEC [4, 9, 10]. However, assessing the degree of differentiation by histopathological criteria may be challenging and consensus is difficult to obtain even among expert pathologists [11, 12]. To help discriminate between these two groups of neoplasms, studies have found mutations in *TP53* or *Rb1* and abnormal p53 and Rb1 immunohistochemical staining, as well as *KRAS* and *BRAF* mutations, to be a feature of NEC; whereas immunohistochemical loss of DAXX/ATRX, mutations in *MEN1* and immunohistochemical expression of CgA and SSTR-2a is more frequently found in pancreatic NET G3 [7, 8, 11–14].

Therefore, it is likely to assume that immunohistochemical stains for p53, SSTR-2a and CgA holds prognostic information for GEP-NEN with a Ki67-index > 20%, but whether this actually is the case remains to be clarified.

We hypothesize that abnormal p53 expression is associated with shorter overall survival (OS), and that high expression of SSTR-2a and CgA is associated with longer OS.

The aim of the present study is, in an in-depth analysis, to elucidate to which degree immunohistochemical expression of SSTR-2a, CgA and p53 is present, and whether these markers can be used as prognostic tools and for risk-stratification of GEP-NEN with a Ki67-index > 20%.

## Methods

### Inclusion and exclusion criteria

Patients with GEP-NEN with a Ki67-index > 20% diagnosed between the 1st of January 2012 and 31st of December 2016 and referred to the ENETS Neuroendocrine Tumour Centre of Excellence, Rigshospitalet were included. Patients with neuroendocrine cancer of unknown primary (CUP) predominantly with abdominal metastases were also included, in agreement with several prior publications [6, 10, 15–20].

Appendiceal goblet cell carcinoids and mixed neuroendocrine non-neuroendocrine neoplasms (MiNEN), were excluded. A total of 163 patients were included in the study.

### Data collection

Basic patient characteristics, data on blood biochemistry, pathology, clinical information, outcome and survival were retrospectively recorded from the patients’ files and from the prospective NEN database at Rigshospitalet (approved by The Danish Data Protection Agency, j.nr. 2007-58-0015).

Clinical data on gender, age, date of diagnosis, WHO performance status (PS), location of primary tumour and TNM-classification (UICC, 7th edition) at time of diagnosis were extracted, as well as data on treatment on date of diagnosis until end of follow-up. These included; surgery (type of operation and radicality), chemotherapy (neoadjuvant, adjuvant or palliative chemotherapy and type of chemotherapy), and information about radiotherapy and peptide receptor radionuclide therapy (PPRT). Biochemical data extracted include plasma CgA and serum lactate dehydrogenase (LDH) < 6 months after diagnosis. Date of death was recorded to estimate overall survival (OS).

### Pathology and immunohistochemistry

Data was obtained from the Danish National Pathology Database and included grade (Ki67 proliferation index), pTNM, large vs. small cell morphology, tumour differentiation (poorly differentiated NEC vs. well-differentiated NET G3) and the results of IHC-staining for synaptophysin, CgA, SSTR-2a (UMB-1) and p53.

All tumours were reclassified according to the WHO 2019 classification. Tumours were classified as poorly differentiated when presenting with marked nuclear atypia, geographical necrosis and a desmoplastic stroma, and classified as well-differentiated, when presenting with a typical neuroendocrine organoid growth pattern (nested, trabecular, glandular etc.) without geographic necrosis and with low-grade cytological atypia, a regular capillary pattern and a fibrotic or hyalinised stroma [8, 21, 22].

Ninety-two cases (56%) were biopsies and 71 cases (44%) were resected tumour specimens.

Formalin fixed, paraffin-embedded samples of representative tumour specimens were selected for immunohistochemical studies and performed on 3–4 μm thick sections. For details of antibodies and immunohistochemical methods, please see “Immunohistochemistry” in Appendix.

Ki67 proliferation index is expressed as the percentage of 500–2000 cells in areas of highest nuclear labeling with the MIB1-antibody. In biopsies with fewer cells, all cells were counted [3].

**Fig. 1:**
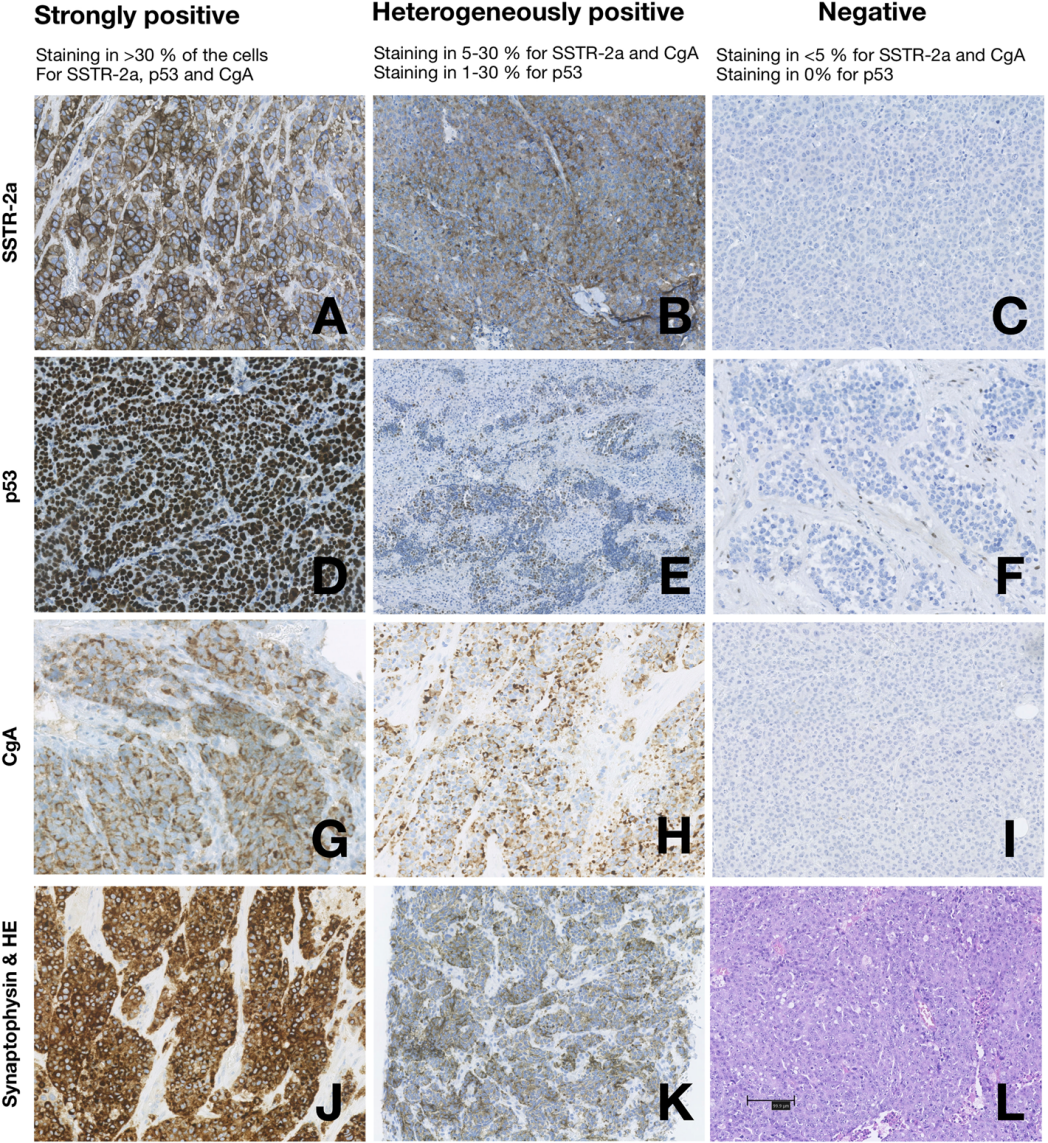
Semiquantitative scoring of SSTR-2a, p53 and chromogranin A immunostaining. Immunohistochemistry in poorly differentiated GEP-NEC of membranous SSTR-2a expression (**a**-**c**), nuclear p53-expression (**d-f**), cytoplasmic CgA (**g-i**) and cytoplasmic synaptophysin (**j-k**) as well as a haematoxylin eosin section (**l**). A positive internal control with staining of non-neoplastic cells such as lymphocytes, fibroblasts or endothelial cells is shown in the negative p53-staining (**f**). (**e**) and (**h**) shows small cell morphology, whereas (**a-d, f-g** and **i-l**) are large cell tumours. (**a, f, h** and **j**) are from colon sigmoideum, (**b**-**d**, **i** and **k-l**) are from rectum, (**e**) is from a lymph node (CUP), (**g**) is from the oesophagus and the rest are colorectal neuroendocrine neoplasms. Scale bar at (**l**) = 100 μm. Copyright© 2018 the Department of Pathology, Rigshospitalet. All rights reserved

Nuclear (p53), cytoplasmic (synaptophysin, CgA), and membranous (SSTR-2a) staining were scored as specified. SSTR-2a, CgA and p53-immunostaining were assessed using a semi-quantitative scoring system (Fig. 1) in accordance with the classification used at ENETS Neuroendocrine Tumor Centre of Excellence, Rigshospitalet. Cases were scored as strongly positive when staining for CgA and SSTR-2a was seen in > 30% of the tumour cells, as heterogeneously positive when staining was seen in 5–30% independent of staining-pattern (diffuse or focal) or as negative when staining was positive in < 5% of the tumour cells. P53 was scored as negative (0%), heterogeneously positive (1–30%) and strongly positive (> 30%). Strongly positive p53 was considered abnormal and indicated mutations in the *TP53* gene. Negative p53-staining was also considered abnormal in accordance with the scoring system used at other institutions [7].

### Statistical analyses

All statistical analyses were performed using IBM SPSS version 25.00 statistical software (SPSS, Chicago, IL, USA). *P*-values of < 0.05 were considered statistically significant.

The primary endpoint was OS, defined as the time from the date of diagnosis to the date of death. At the end of follow up, on the 10th of February 2018, the remaining cases were censored.

Patient characteristics, histopathological characteristics and expression of SSTR-2a, p53, CgA and Ki67 were factors analysed to determine effect on the primary endpoint. Kaplan-Meier plots and the log-rank test were used for OS analyses. Hazard ratios (HRs) with 95% confidence intervals (CI) and *p*-values were estimated using Cox proportional hazard model. Variables clinically or statistically relevant (*p*-value of < 0.05) in the univariate analysis were selected, dichotomised and introduced in the multivariate model: Age (> 65 years), gender, grade of differentiation (well- vs. poorly differentiated), primary tumour (pancreas vs. other primary tumours), tumour burden (locoregional disease vs. disseminated), performance status (PS > 2), and treatment (surgery or chemotherapy).

The three groups (negative, heterogeneously positive and strongly positive) for SSTR-2a, CgA and p53 were dichotomised in different combinations for subgroup analysis and the multivariate analysis. Continuous variables are reported as median and range for age and Ki67. Categorical variables of IHC, tumour and patient characteristics were correlated by Chi-square test or Fisher’s exact test when appropriate. Independence-samples T test was used to compare means of two groups. One-way ANOVA was used for comparison of 3 or more groups with the Tukey HSD test for Post Hoc groupwise comparison.

## Results

### Patient characteristics

Between January 2012 and December 2016, 163 patients with GEP-NEN or CUP with predominant abdominal metastases and with a Ki67-index > 20% were included (Table 1). Patients had a median age of 69 years (range 23–89 years), and 42% were females. The majority of patients (*n* = 112, 69%) had distant metastases at diagnosis.

**Table 1:** Baseline and clinical characteristics in 163 patients

Variable	N	(%)
Age: median (years), range	69	(23–89)
Gender: female/male	68/95	(42%/58%)
Primary tumour		
Upper GI	38	(23%)
Oesophagus	10	(6%)
Gastrooesophageal junction (GEJ)	15	(9%)
Stomach	13	(8%)
Pancreas	28	(18%)
Pancreas	28	(18%)
Lower GI	64	(39%)
Small intestine	3	(2%)
Colorectal	61	(37%)
Cancer unknown primary (CUP) (predominantly with abdominal metastases)	33	(20%)
Extension of disease:		
Localised	15	(9%)
Regional	36	(22%)
Disseminated	112	(69%)
WHO Performance Status	147	(90%)
0	61	(41%)
1	48	(33%)
2	22	(15%)
> 2	16	(11%)
Treatment	142	(87%)
Surgery	71	(44%)
Primary tumour	61	(86%)
Metastasis	19	(27%)
Operation at diagnosis	56	(79%)
Curative intent	53	(75%)
Chemotherapy	119	(73%)
Neoadjuvant chemotherapy	14	(12%)
Adjuvant chemotherapy	28	(24%)
Palliative chemotherapy	103	(87%)
Radiotherapy^a^	46	(28%)
Primary tumour	6	(13%)
Metastasis	41	(89%)
PRRT	2	(1%)
Lactate dehydrogenase (LDH), serum level	143	(88%)
Normal	67	(47%)
< 2 UNL^b^	52	(36%)
> 2 UNL	24	(17%)
Chromogranin A (CgA), plasma level	112	(69%)
Normal	61	(55%)
< 2 UNL	20	(18%)
> 2 UNL	31	(28%)

^a^ One patient was treated with radiotherapy for both the primary tumour and the metastases ^b^ UNL = upper normal limit of the reference value

WHO PS was available for 147 patients, and 109 (74%) had PS 0–1. One hundred forty-two patients (87%) received treatment. The remaining 13% received best supportive care due to poor PS or due to abstaining from treatment.

Chemotherapy was given to 119 patients (73%), and 71 (44%) underwent surgery. Of these 71 operated patients 61 (86%) had the primary tumour and regional lymph nodes resected. Nine of 84 patients with liver metastases underwent liver resection (*n* = 6) or radiofrequency ablation (*n* = 3). Of the 71 operated patients, 49 had neoadjuvant, adjuvant and/or palliative chemotherapy administered, and 23 of these patients also received radiotherapy. Of the 53 patients operated with curative intention, R0 (*n* = 45) or R1 (*n* = 8) resection was achieved at the first operation.

Surgery was most often performed for primaries in the upper or lower gastrointestinal (GI) tract compared to pancreatic primaries and CUPs: upper GI: 47% (*n* = 18/38) and lower GI 69% (*n* = 44/64) vs. pancreas 25% (*n* = 7/28) and CUP 6% (*n* = 2/33).

### Tumour characteristics in 163 high grade GEP-NEN patients

Examples of strongly positive, heterogeneously positive and negative immunostaining for SSTR-2a, p53, CgA and synaptophysin are presented in Fig. 1.

All 163 tumours were immunoreactive for synaptophysin, 107 (66%) for chromogranin A and the median Ki67-index was 90% (range: 21–100%) (Table 2).

**Table 2:** Tumour pathology and immunohistochemistry in 163 patients

Variable	N	(%)
Proliferation index	163	(100%)
Ki67: median (%), range	90	(21–100)
Morphology	163	(100%)
NET G3, Well-differentiated (all large cell)	14	(9%)
NEC, Poorly differentiated	149	(91%)
Small cell NEC (SCNEC)	21	(14%)
Large cell NEC (LCNEC)	128	(86%)
p53	162	(100%)
Negative (0%)	50	(31%)
Heterogeneously positive (1–30%)	32	(20%)
Strongly positive (> 30%)	80	(49%)
SSTR-2a	163	(100%)
Negative	101	(62%)
Heterogeneously positive	19	(12%)
Strongly positive	43	(26%)
Chromogranin A	163	(100%)
Negative	56	(34%)
Heterogeneously positive	40	(25%)
Strongly positive	67	(41%)
Synaptophysin	163	(100%)
Heterogeneously positive	2	(1%)
Strongly positive	161	(99%)

Fourteen tumours were well-differentiated, relatively more frequent in pancreatic primaries (21%, *n* = 6/28; lower GI 8%, *n* = 5/64; upper GI 5%, *n* = 2/38; CUP 3%, *n* = 1/33) (*p* = 0.08).

Immunohistochemistry was performed on all 163 tumours and presented in Table 2. One test for p53 was missing due to lack of remaining tissue.

### Origin of primary tumour in relation to p53, SSTR-2a and CgA

**Fig. 2a-c:**
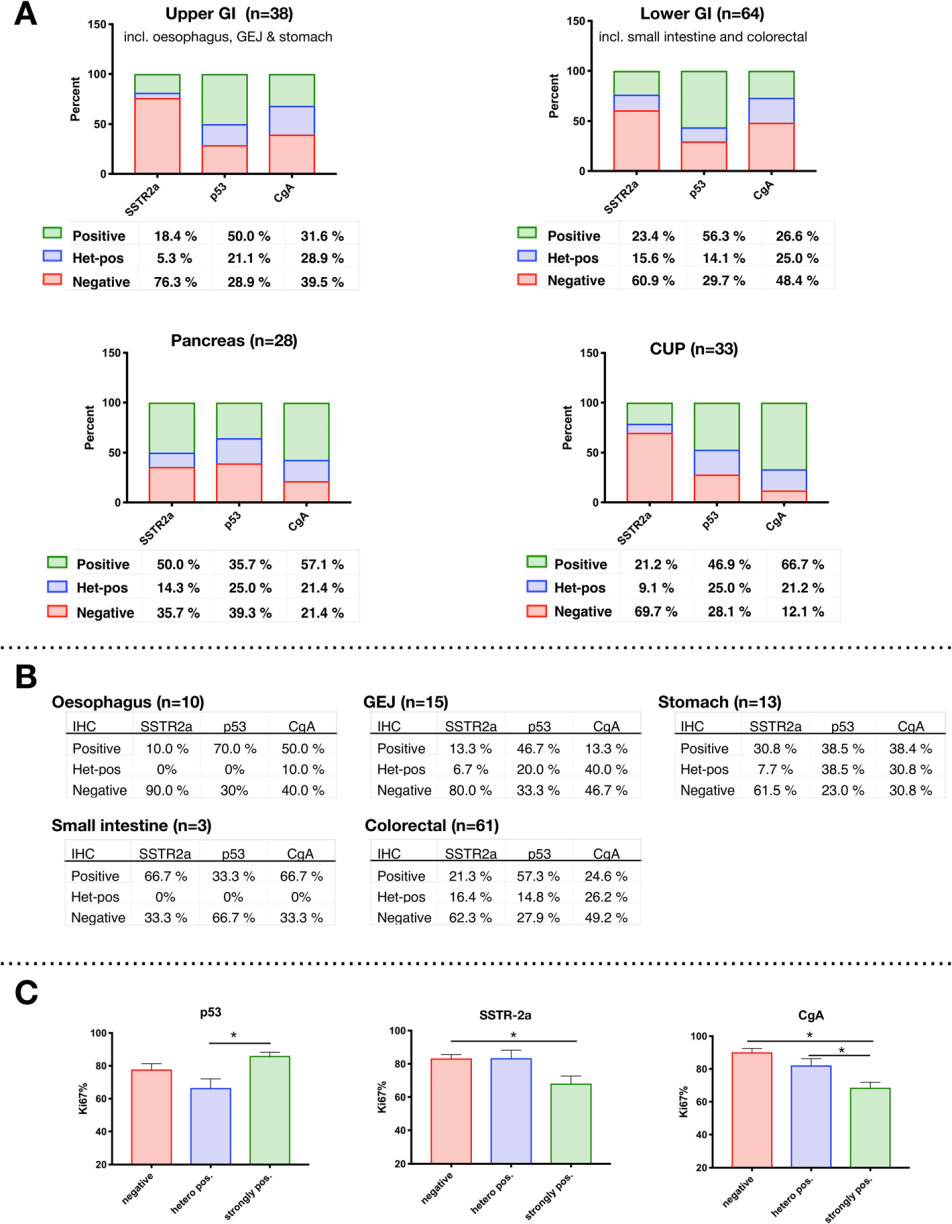
The immune-markers SSTR-2a, p53 and CgA in relation to origin of primary tumour and Ki67. **a**: Percentages for each marker is shown beneath each bar. For SSTR-2a, p53 and CgA the number of patients for upper GI is 38, for lower GI 64, for pancreas 28 and for CUP 33; except for p53, which are 32 due to one missing analysis. Differences in distribution of the immune-markers are visualised according to primary tumour. **b**: The distribution of immunomarkers for the specific localisation of the primary tumor is presented. **c**: The Ki67-index compared to the staining pattern of p53, SSTR-2a and CgA

Primary tumour origin in relation to p53, SSTR-2a and CgA is presented in Fig. 2a and b. A significant difference in distribution of the staining pattern was found for CgA (*p* = 0.001) and SSTR-2a (*p* = 0.028), but not for p53 (*p* = 0.590).

Upper and lower GI tumours had a large proportion of negative SSTR-2a (upper GI: 76%; lower GI: 61%), negative CgA (upper GI: 40%; lower GI: 48%), and strongly positive p53-staining (upper GI: 50%; lower GI: 56%). Strongly positive SSTR-2a (50%) and CgA-expression (57%) was more than twice as frequent in pancreatic primaries compared to upper GI (18 and 32%, respectively) and lower GI (23 and 27%, respectively). Furthermore, strongly positive p53-staining was less often present in pancreatic primaries (36%) in contrast to the other primaries, while heterogeneously positive and negative p53-staining was more frequent in pancreatic primaries.

In patients with cancer of unknown primary, a large proportion of tumours were strongly positive for CgA (67%), and negative for SSTR-2a (70%). p53 was overexpressed in 47% of the CUPs.

### Correlation between the p53, SSTR-2a, CgA, differentiation, Ki67 and cell type

The association between distributions of the three immunohistochemical markers p53, SSTR-2a and CgA is shown in Table 3.

**Table 3:** Immunohistochemistry and tumour characteristics in p53, SSTR-2a and CgA

p53	Total, rows	Negative	Het-pos.	Positive	*p*
	*n* = 162 (100%)	*n* = 50 (30.9%)	*n* = 32 (19.8%)	*n* = 80 (49.4%)	
SSTR-2a Negative	101 (62.3%)	29 (17.9%)	12 (7.4%)	60 (37.0%)	0.01*
SSTR-2a Het-pos.	19 (11.7%)	5 (3.1%)	7 (4.3%)	7 (4.3%)	
SSTR-2a Positive	42 (25.9%)	16 (9.9%)	13 (8.0%)	13 (8.0%)	
NET G3, Well diff.	14 (8.6%)	6 (3.7%)	5 (3.1%)	3 (1.9%)	0.05*
NEC, Poorly diff.	148 (91.4%)	44 (27.2%)	27 (16.7%)	77 (47.5%)	
Small cell	21 (13.0%)	8 (4.9%)	5 (3.1%)	8 (4.9%)	0.53
Large cell	141 (87.0%)	42 (25.9%)	27 (16.7%)	72 (44.4%)	
SSTR-2a	Total, rows	Negative	Het-pos.	Positive	*p*
	*n* = 163 (100%)	*n* = 101 (62.0%)	*n* = 19 (11.7%)	*n* = 43 (26.4%)	
CgA Negative	56 (34.4%)	45 (27.6%)	7 (4.3%)	4 (2.5%)	< 0.001*
CgA Het-pos.	40 (24.5%)	28 (17.2%)	3 (1.8%)	9 (5.5%)	
CgA Positive	67 (41.1%)	28 (17.2%)	9 (5.5%)	30 (18.4%)	
NET G3, Well diff.	14 (8.6%)	7 (4.3%)	0 (0%)	7 (4.3%)	0.08
NEC, Poorly diff.	149 (91.4%)	94 (57.7%)	19 (11.7%)	36 (22.1%)	
Small cell	21 (12.9%)	11 (6.9%)	3 (1.8%)	7 (4.3%)	0.54
Large cell	142 (87.1%)	90 (55.2%)	16 (9.8%)	36 (22.1%)	
CgA	Total, rows	Negative	Het-pos.	Positive	*p*
	*n* = 163 (100.0%)	*n* = 56 (34.6%)	*n* = 40 (24.7%)	*n* = 67 (40.7%)	
p53 Negative	50 (30.9%)	19 (11.7%)	7 (4.3%)	24 (14.8%)	0.03*
p53 Het-pos.	32 (19.8%)	6 (3.7%)	9 (5.6%)	17 (10.5%)	
p53 Positive	80 (49.4%)	31 (19.1%)	24 (14.8%)	25 (15.4%)	
NET G3, Well diff.	14 (8.6%)	1 (0.6%)	3 (1.8%)	10 (6.1%)	0.03*
NEC, Poorly diff.	149 (91.4%)	55 (33.7%)	37 (22.7%)	57 (35.0%)	
Small cell	21 (12.9%)	12 (7.4%)	4 (2.5%)	5 (3.1%)	0.07
Large cell	142 (87.1%)	44 (27.0%)	36 (22.1%)	62 (38.0%)	

Fischer’s exact test, *p*-value. *Statistically significant with a *p*-value < 0.05

A large proportion of tumours stained negative for SSTR-2a and strongly positive for p53 (37%, *n* = 60/162), which was the most frequent staining combination. Negative staining for both SSTR-2a and p53 was seen in 18% of cases (*n* = 29/162). For CgA, the majority of negatively stained tumours were also negative for SSTR-2a, 28% (*n* = 45/163). Tumours stained strongly positive for both CgA and SSTR-2a in 18% of the cases (*n* = 30/163). Furthermore, 19% of all cases (*n* = 31/162) were CgA negative and strongly positive for p53.

The distribution of tumour differentiation in relation to immunohistochemical markers differed significantly for CgA (*p* = 0.03) and p53 (*p* = 0.05), but not for SSTR-2a (*p* = 0.08) (Table 3). Fourteen tumours (9%) were well-differentiated NET G3. Well-differentiated morphology was most frequent in heterogeneously stained p53 tumours (16%, *n* = 5/32), followed by negative p53 (12%, *n* = 6/50) and less frequent in strongly positive p53 tumours (4%, *n* = 3/80). For CgA, well-differentiated morphology was found in 15% (*n* = 10/67) of the strongly positive group, 8% of the heterogeneously positive group (n = 3/40) and 2% of the negative group (n = 1/56). For SSTR-2a, well-differentiated morphology was present in 16% of the strongly positive (*n* = 7/43) compared to 7% of the negative (n = 7/101) and none of the heterogeneously positive tumours.

The mean Ki67-index with standard deviation was significantly different between NET G3 (42.5 +/− 24.0) and NEC (82.8 +/− 22.7) (*p* < 0.0001).

By one-way ANOVA there was a significant relation between the three markers and the Ki67-index (p53 *p* = 0.001; SSTR-2a *p* = 0.003 and CgA *p* < 0.001) (Fig. 2c). For p53, the mean Ki67-index was significantly lower in the heterogeneously positive compared to the strongly positive tumours (Tukey HSD, *p* = 0.001). Tumours staining strongly positive for SSTR-2a had a significantly lower Ki67-index than negatively stained tumours (*p* = 0.003), while there was no significant difference in Ki67-index between strongly positive and heterogeneously positive tumours (*p* = 0.066). Tumours staining strongly positive for CgA had a significantly lower Ki67-index than the heterogeneously positive (*p* = 0.012) and negative tumours (*p* < 0.001). The Ki67 index was not significantly different between the negatively stained and heterogeneously positive tumours for any of the three markers.

Twenty-one tumours were small-cell NEC. There was no statistically significant difference in the distribution of small- and large cell morphology in relation to the expression of the immunohistochemical markers.

### Survival and general prognostic factors in the entire cohort

Median follow up time was 42.0 months (range 0.5–82 months). On the 10th of February 2018, 130 patients had died. Median overall survival (OS) in the entire cohort was 11.0 months (range 0.5–82.0 months) and the cumulative overall survival rate was 48% at 1 year, and 19% at 3 years.

**Fig. 3:**
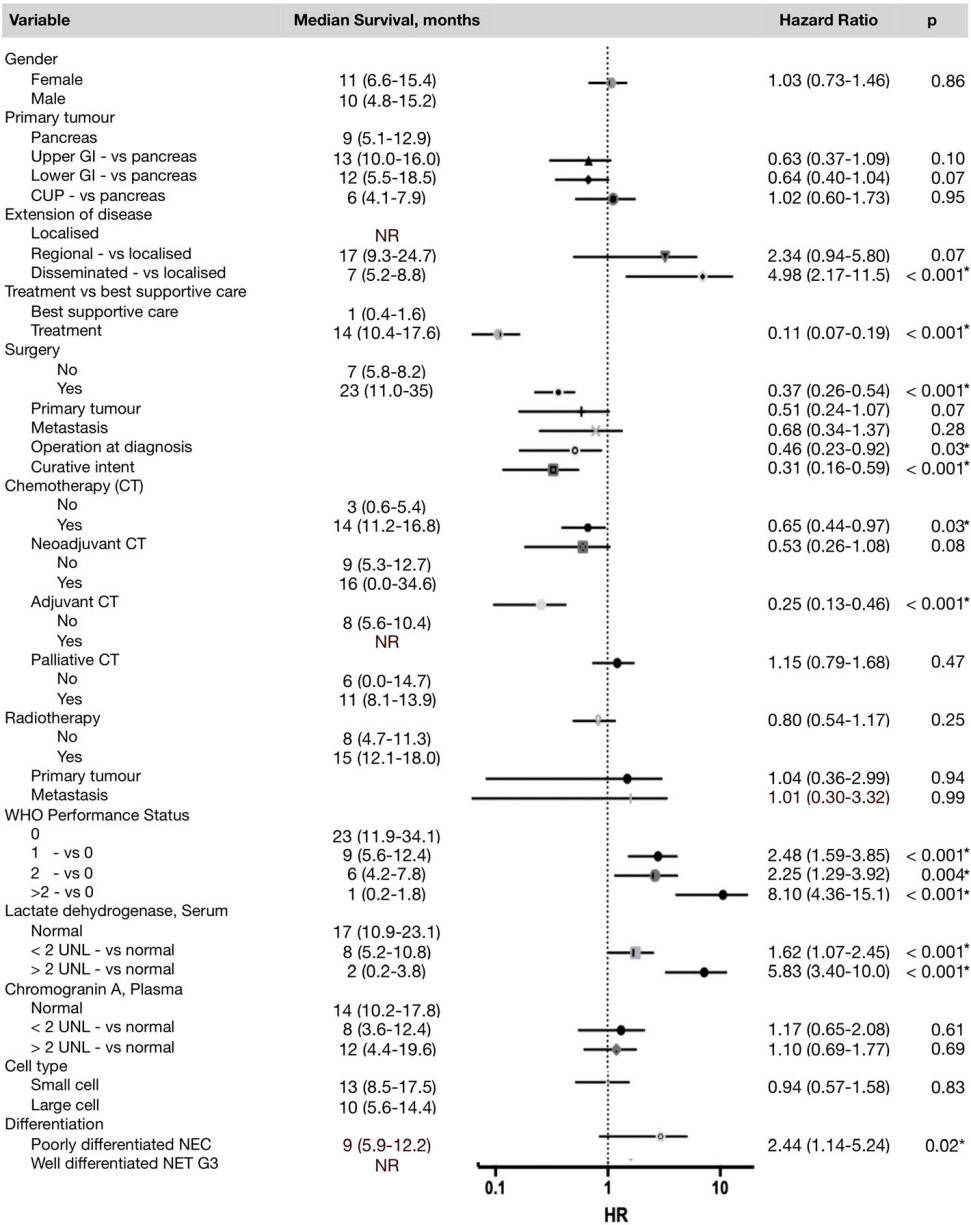
Univariate survival analyses of prognostic parameters in 163 patients. Median survival in months are shown for each parameter, as well as HRs with CI and *p*-values. The 95% confidence intervals are shown as horizontal lines. CI’s crossing the line at HR = 1 are non-significant. The dot on each line indicates the HR. NR = not reached. *Indicates significant *p*-values < 0.05

In the univariate cox regression analysis (Fig. 3), disseminated disease (*p* < 0.001), poor performance (PS > 2; *p* < 0.001), elevated serum LDH level (LDH > 2 UNL; *p* < 0.001), and poorly differentiated morphology (*p* = 0.02) were associated with poor survival. Any treatment (*p* < 0.001), surgery (*p* < 0.001), chemotherapy (*p* = 0.03) and adjuvant chemotherapy (*p* < 0.001) was associated with improved survival.

Despite the fact that the primary tumour origin did not reach statistical significance, there was a tendency for differences between the groups: Pancreatic primaries and CUPs had a median survival of 9 (5.1–12.9) and 6 (4.1–7.9) months, respectively, compared to upper and lower GI primaries with a median survival of 13 (10.0–16.0) and 12 (5.5–18.5) months, respectively.

### The prognostic role of p53, SSTR-2a and CgA in univariate and adjusted multivariate analyses

**Fig. 4:**
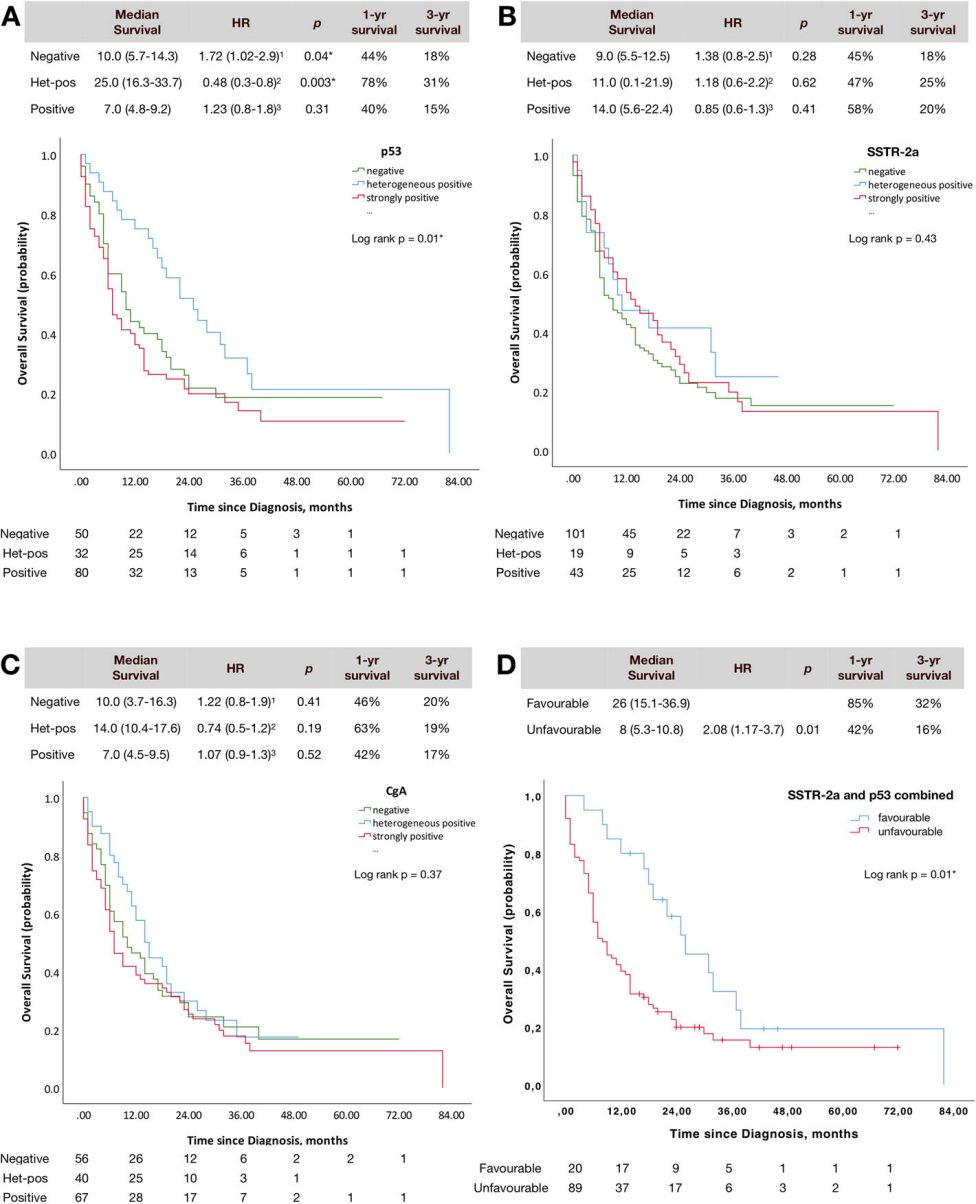
Univariate analysis for p53 (**a**), SSTR-2a (**b**), CgA (**c**) and in combination (**d**). Kaplan-Meier Plots with number at risk, median survival (CI), hazard ratio (CI) and *p*-value, 1-yr and 3-yr overall survival, and log-rank p-value for correlation of all three immune-scores in the entire group. All hazard ratios are calculated pair wise as: 1: negative (vs. het-pos.), 2: het-pos (vs. positive), 3: positive (vs. negative). **d** shows a sub analysis for p53 and SSTR-2a in combinations, where the hypothetically favourable profile is SSTR-2a strongly positive or het-pos with p53 het-pos compared to the unfavourable profile with SSTR-2a negative and p53 negative or strongly positive

Results from the univariate analyses are presented in Figs. 4a-d. Kaplan Meier plots with log-rank *p*-values, median survival, 1- and 3-year survival and HRs with *p*-values have been estimated for p53 (Fig. 4a), SSTR-2a (Fig. 4b) and CgA (Fig. 4c). Figure 4d shows a combination of p53 and SSTR-2a. Multivariate analyses are presented in Table 4.

**Table 4:** Adjusted multivariate survival analyses

Comparison of staining	p53		SSTR-2a		CgA	
	HR (CI)	*p*	HR (CI)	*p*	HR (CI)	*p*
All 3 categories without dichotomisation	1.26 (0.99–1.60)	0.07	0.91 (0.72–1.15)	0.41	0.89 (0.69–1.15)	0.38
A: Neg (vs. het-pos)	1.88 (1.01–3.49)	0.05*	1.18 (0.61–2.29)	0.63	2.11 (1.15–3.88)	0.02*
B: Het-pos (vs. pos)	0.33 (0.19–0.60)	< 0.001*	1.18 (0.54–2.58)	0.68	1.13 (0.68–1.87)	0.63
C: Pos (vs. neg)	1.30 (0.83–2.04)	0.25	0.84 (0.52–1.35)	0.47	0.78 (0.47–1.30)	0.34
D: Neg (vs. het-pos + pos)	1.03 (0.67–1.58)	0.88	1.19 (0.78–1.82)	0.41	1.39 (0.89–2.18)	0.15
E: Het-pos (vs. neg + pos)	0.45 (0.27–0.75)	0.002*	1.08 (0.57–2.04)	0.82	1.31 (0.84–2.04)	0.23
F: Pos (vs. het-pos + neg)	1.85 (1.24–2.76)	0.003*	0.84 (0.53–1.34)	0.47	0.97 (0.65–1.47)	0.89

Adjusted for age (> 65 years), gender, grade of differentiation (well- vs. poorly differentiated), primary tumour (pancreas vs. other primary tumours), tumour burden (locoregional disease vs. disseminated), performance status (PS > 2), and treatment (surgery or chemotherapy). The immune-staining outside the parenthesis in the column “Comparison of staining” indicated which score the HR relates to. *Indicates significant *p*-values < 0.05. Unadjusted HR (CI) and *p*-values are presented in Fig. 4, and were significant for p53 in the A and B comparison in the univariate analyses

### p53 as a prognostic marker

The median survival for the different scores of p53 is shown in Fig. 4a. Median survival was 25 (16.3–33.7) months for heterogeneously positive p53. This was more than twice as long compared to the negative group with a median survival of 10 (5.7–14.3) months, and the strongly positive group with a median survival of 7 (4.8–9.2) months. Accordingly, the heterogeneously positive group had a better prognosis compared to the strongly positive (*p* = 0.003), and the negative p53 (*p* = 0.04) in the univariate analysis. No significant prognostic difference was found between the p53 negative and strongly positive groups (*p* = 0.31).

Survival rates in patients with a heterogeneously positive p53-score was 78% at 1 year and 31% at 3 years, compared to those with a negative or strongly positive p53-score having survival rates of 44 and 40% at 1 year, and 18 and 15% at 3 years, respectively.

In the adjusted multivariate analyses (Table 4), strongly positive p53 was shown to be an independent negative prognostic factor, when compared to patients with a negative (*p* = 0.05) or heterogeneously positive p53-expression (*p* < 0.001), confirming the findings of the univariate analysis. A significantly better outcome was also seen, when normal p53 was defined as heterogeneously positive and abnormal p53 was defined as both strongly positive and negative (*p* = 0.002). Sub-analyses comparing the three groups one by one showed that strongly positive p53 has a worse outcome than both negative and heterogeneously positive p53 (*p* = 0.003).

### SSTR-2a as a prognostic marker

There was a tendency towards longer survival amongst patients with increasingly positive tumour-expression of SSTR-2a, although not statistically significant (*p* = 0.43) (Fig. 4b).

By the method of Kaplan-Meier, SSTR-2a expression did not hold any prognostic value, neither when categorised as negative, heterogeneously positive and strongly positive, nor when dichotomised above or below 30% positive SSTR-2a immunostaining.

When stratified for origin of primary tumour, a correlation was seen in the pancreatic NEN (*p* = 0.02). Due to the small number of patients with pancreatic primaries (*n* = 28), the three groups were dichotomised as negative and heterogeneously positive vs. positive. Improved survival (*p* = 0.02) was seen for the SSTR-2a positive group with a median survival of 14 (2.2–25.8) months vs. 7 (5.2–8.8) months for the negative/heterogeneously positive group.

In multivariate analyses, the combination of heterogeneously positive and strongly positive SSTR-2a was not an independent positive prognostic indicator (*p* = 0.34). Neither were any of the combinations presented in Table 4.

### CgA as a prognostic marker

Median survival was 10.0 (3.7–16.3), 14.0 (10.4–17.6) and 7.0 (4.5–9.5) months for negative, heterogeneously positive and positive CgA expression, respectively (*p* = 0.37).

The Kaplan-Meier analysis did not show any significant associations with increased survival between the groups (CgA negative, CgA het.pos. and CgA strongly positive) (Fig. 4c).

In multivariate analyses, the combination of heterogeneously positive and strongly positive CgA were not independent positive prognostic indicators (*p* = 0.12).

However, negative CgA was associated with a significantly worse OS compared to heterogeneously positive CgA-expression (*p* = 0.02) (Table 4).

### Prognostic value of combined p53 and SSTR-2a

A sub-analysis was performed combining p53 and SSTR-2a. We hypothesised that a favourable profile of strongly positive or heterogeneously positive SSTR-2a combined with p53 heterogeneously positive (*n* = 20) would be associated with longer survival than an unfavourable profile with SSTR-2a negative combined with p53 negative or strongly positive (*n* = 89) (Fig. 4d). The combination of SSTR-2a and p53 was significantly associated with better survival (*p* = 0.02). The median overall survival of 26 (19.7–32.3) months with the favourable profile is more than three times as long compared to 8 (5.3–10.8) months for the unfavourable profile. Survival rates were also twice as high in the group with the favourable profile for 1- and 3-year survival with 85 and 32% in the favourable group, and 42 and 16% in the unfavourable group. This was also significant in the multivariate analysis, where an unfavourable profile had a HR (CI) 2.5 (1.3–4.9) with a *p*-value 0.005.

## Discussion

In the present study the prognostic value of the immunohistochemical markers p53, SSTR-2a and CgA were thoroughly investigated in a large cohort of 163 GEP-NEN patients with a Ki67-index > 20%, including well-differentiated NET G3 and poorly differentiated NEC according to the recent WHO 2019 classification.

Absent and strongly positive p53 expression was considered abnormal. Abnormal p53 was an independent negative prognostic factor suggesting its use for risk stratification in these highly malignant tumours with a median survival of less than 10 months compared to patients with normal p53, whose median survival was 25 months. Strongly positive p53-expression was also an independent negative prognostic factor in the multivariate sub-analysis.

Strongly positive SSTR-2a was observed in 26% of the patients, and was shown to be a significant positive prognostic marker in pancreatic NEN with a Ki67-index > 20%. Negative CgA was associated with a significantly worse OS compared to heterogeneously positive CgA-expression in a multivariate sub-analysis.

The mean Ki67-index was significantly different between well-differentiated NET G3 and NEC (*p* < 0.0001). Well-differentiated tumours was likely to be associated with heterogeneously positive p53, as well as strongly positive SSTR-2a and CgA, and was more frequently observed in pancreatic primaries.

Furthermore, the mean Ki67-index was significantly lower in tumours staining heterogeneously positive for p53, and strongly positive for CgA and SSTR-2a. As patients with lower Ki67-index has an improved survival compared to patients with a high Ki67-index [1], the findings indirectly substantiate that abnormal p53 is associated with poor survival and high expression of SSTR-2a and CgA is associated with improved survival, as also found in this study.

In agreement with previous studies, we found advanced disease, poor PS, elevated serum LDH and poorly differentiated morphology to be significantly associated with worse survival.

In contrast, surgery and chemotherapy, in particular adjuvant therapy, was associated by improved survival [1, 16].

Protein expression of p53 assessed by IHC is a surrogate reflecting the underlying *TP53* mutation status of a tumour, and has utility in the diagnostic and prognostic workup of many cancers [23]. Since there has not been any consensus of interpretation and cut-offs in NEN regarding the immunostaining, especially regarding p53, abnormal p53-expression was defined as complete absent (0%) or strongly positive (> 30%). Abnormal p53-expression was associated with shorter survival and both the negative and strongly positive p53 tumours had a worse prognosis compared to the heterogeneous p53-expression in uni- and multivariate analyses. More studies have proposed a definition of abnormal p53 as “all or nothing”, i.e. overexpressed or completely absent [7, 23–26].

In the study by Konukiewitz et al. [7] of pancreatic and extrapancreatic NEN G3 according to the WHO 2017 classification, p53-expression was scored as abnormal in cases of moderate to strong nuclear positivity in more than 20% of the cells or in cases of complete absence. Abnormal p53-expression and *TP53* alterations were restricted to poorly differentiated neuroendocrine neoplasms, and presence of *TP53* mutations correlated, in 92% of the cases, with abnormal p53-immunolabeling [7].

Ali et al. [27] studied the prognostic value of p53 in GEP-NEC patients. The p53-immunoreactivity was defined as positive when staining in > 10% of the tumour cells and it was found that p53-immunoreactivity was correlated with poor OS in patients with colorectal tumours with distant metastases, but not in the entire cohort of 124 GEP-NEC patients, nor in the pancreatic primaries. This discrepancy between the present study and the study by Ali et al. might be explained by differences in interpretation of p53-staining as normal and abnormal, as well as differences in arbitrary cut-off points. These interpretational difficulties are reflected in several prior studies, not restricted to neuroendocrine neoplasms [7, 25, 28, 29]. Therefore, it is necessary to have consensus-guidelines regarding cut-offs if p53-immunohistochemistry is to be used as a tool in the classification of NET G3 and NEC.

Overall, SSTR-2a expression was not a prognostic marker in this cohort. However, a strong immunohistochemical expression of SSTR-2a was an independent positive prognostic marker in pancreatic tumours, most likely indicating a more well-differentiated tumour. The prognostic significance has primarily been studied in NET G1 and G2. In a large prospective trial of 279 NET patients, SSTR-2a IHC proved to be an independent prognostic marker for overall survival and confirmed that SSTR-2a IHC correlated with SSTR2-imaging [30]. Another study including 99 pancreatic NET also found expression of SSTR-2a to be an independent positive prognostic factor for survival even superior to Ki67 [31].

SSTR-2a is believed to be an indicator of well-differentiated morphology, and the discriminative significance of the surrogate marker SSTR-2a and p53 have recently been studied in pancreatic NEN with a Ki67-index > 20% [7]. We found that SSTR-2a expression is not restricted to well-differentiated tumours as it was detected as strongly positive in 26% and heterogeneously positive in 12% of the poorly differentiated GEP-NEN, which is confirmed by various studies [7, 12, 14, 32]. The presence of somatostatin receptors makes these neuroendocrine neoplasms potentially amenable to treatment with PRRT in selected patients with high uptake on somatostatin receptor imaging as proposed in ENETS guidelines and in the study by Carlsen et al. [16, 33]. The present study also showed that the combination of SSTR-2a and p53 holds important prognostic information.

The presence of positive staining for CgA is considered to be a good prognostic marker [1, 34]. In our study, immunostaining was strongly positive in 41% of the cases. CgA-expression was not a significant prognostic marker, despite a statistically significant result in a sub-analysis comparing tumours with heterogeneously positive vs. negative CgA-staining. No prognostic significance was found in sub-analysis regarding origin of the primary tumour. This confirms the finding of previous studies [1, 35–37].

Strengths of the present study include the relatively large number of GEP-NEN patients with a Ki67-index > 20%, data extraction from the national pathology database, the combination of tissue markers, all evaluated by experienced GEP-NEN pathologists, clinical data on treatment, and the completeness of follow-up, which facilitated comprehensive survival analyses.

A limitation to the study is the retrospective nature of the study. Staging according to AJCC version 8 would have been preferred, but not possible as patients were staged before 2017. Furthermore, only 14 patients with NET G3 were included. Morphological classification alone may be insufficient as suggested by several other studies [11, 12], and evaluation of *TP53* was not possible.

## Conclusion

To the best of our knowledge, this is the hitherto largest study to determine the prognostic significance of the immunohistochemical markers p53, SSTR-2a and CgA in high grade GEP-NEN, including NET G3 and NEC. The results confirm the findings of recent papers, and provide further evidence that high grade GEP-NEN patients with abnormal p53 have a worse prognosis with less than half the median survival time as patients with normal p53.

The 3 markers were all found to have a significant relation to the Ki67-index.

The relatively most frequent combination of markers observed was a negative SSTR-2a and a positive p53, suggesting that the combination of SSTR-2a and p53 can be used for risk stratification.

SSTR-2a expression was shown to be a significant positive prognostic marker in pancreatic NEN with a Ki67-index > 20%. Negative CgA was associated with a significantly worse OS compared to heterogeneously positive CgA-expression in a multivariate sub-analysis. The conclusion of the study essentially confirms that NEC (as defined by p53 mutation) has a worse prognosis than NET G3.

## Data Availability

The datasets used and/or analysed during the current study are available from the corresponding author on reasonable request.

## Appendix

### Immunohistochemistry

Immunohistochemical evaluation was performed using the Ki67 (clone 30–9), CgA (clone LK2H10) and p53 (clone DO-7) antibodies from Roche, and the synaptophysin (clone DAK-synapt) antibody from Agilent (Dako); in all cases ready-to-use (RTU) kits following the manufacturer’s instructions. To visualise SSTR-2a the monoclonal antibody UMB-1 (clone UMB1, ab134152) from Abcam was used, diluted 1:400. The staining for Ki67, CgA, p53 and UMB-1 took place on the Ventana Benchmark Ultra from Roche utilising the UltraView/Optiview detection kit (760–500/760–700) and were incubated for 12, 24 and 28 min respectively. The sections were counterstained with haematoxylin. For the immunohistochemical evaluation of synaptophysin, the sections were briefly pre-treated in PT Link from Dako using high pH/Low pH target retrieval solution (Dako DM828). The staining took place on the DakoLink 48 from Dako utilising the EnVision Flex+ detection kit (Dako K8002). The primary antibody was incubated for 20 min, and the sections were counterstained with haematoxylin. Positive control tissue was stained in parallel with each case.

## Abbreviations

CgA: Chromogranin A
CI: Confidence intervals
CUP: Cancer of unknown primary
Diff.: Differentiated
G1: Grade 1
G2: Grade 2
G3: Grade 3
GEJ: Gastrooesophageal junction
GEP: Gastroenteropancreatic
GI: Gastrointestinal
HE: Haematoxylin eosin
Het-pos: Heterogeneously positive
HR: Hazard ratio
IHC: Immunohistochemistry
LDH: Lactate dehydrogenase
NEC: Neuroendocrine carcinoma
Neg: Negative
NEN: Neuroendocrine neoplasm
NET: Neuroendocrine tumour
OS: Overall survival
p: *P*-value
PD: Poorly differentiated
PDNEC: Poorly differentiated NEC
Pos: Positive
PRRT: Peptide receptor radionuclide therapy
PS: Performance status
SSTR-2a: Somatostatin receptor 2a
UNL: Upper normal limit of the reference value
WD: Well-differentiated
WHO: World Health Organization
